# Notes and News

**Published:** 1904-11-26

**Authors:** 


### NOTES AND NEWS.

A PASTEUR Institute has been opened in Texas.

THE Danish Government has awarded a pension to the widow of the late Professor Finsen.

SIR JAMES RECKETT has made a donation of £6,000 to the Hull Royal Infirmary to complete the extension of the building.

THE Royal Dental Hospital of London, Leicester Square, has received a legacy of £500, bequeathed by the late George Willis Penson, Esq., formerly for many years a trustee of the charity.

A "DICKENS BALL" is being organised by Lady Maud B. Wilbraham, which will take place early in the year at the Empress Rooms, Royal Palace Hotel, Kensington. The proceeds will be devoted to the West Ham Hospital.

IT is proposed to establish a Consumption Sanatorium for Dumfriesshire, and the scheme met with influential support at a meeting held at Dumfries recently, when Mr. William Younger promised to support the movement by a donation of £500.

MRS. ERNEST HART, widow of the late editor of the *British Medical Journal*, is a lady of great energy and versatility of gifts. She is the editress of an interesting periodical entitled "The House Beautiful," which concerns itself with all appertaining to the structure, maintenance, and embellishment of the home, and contains notes and articles on many subjects of interest at the moment. It is obvious that Mrs. Hart does not forget her medical experience and sympathies, for in the number of the journal before us, under the heading of "Domestic and Personal Hygiene," is a useful article on "Catching Cold," which commences with the pathological aspect of the cold, and proceeds to point out the danger of the ailment and the me ns to avoid it.

THE Hunterian Society intends to dispose of its library.

PROFESSOR OSLER, the new Regius Professor of Medicine at Oxford, is to be honoured at Baltimore by the establishment of a memorial medical and library building.

THE Duke of Portland has given £500 to the Mansfield Hospital towards the enlargement, and the local colliery owners have promised £1,700 for the same purpose.

A NEW wing has been opened at the Dunfermline Cottage Hospital, erected from funds contributed by the family of the late Mr. George Lauder, to establish a memorial to him.

DR. MITCHELL BRUCE has been elected consulting physician at Charing Cross Hospital on his retirement from the active staff. Dr. Mitchell Bruce has been connected with the hospital and medical school for 30 years.

A SUM of nearly £1,000 has already been promised to build a pavilion for consumption at the Northampton General Infirmary. The question of site and maintenance is not yet definitely decided, as a diversity of opinion exists on these points.

A MEMBER of the Board of Management of the Royal Hospital for Diseases of the Chest has promised £2,000 towards the building of a new nurses' home and the addition of sanitary buildings, provided a further £3,000 is forthcoming by the end of June next.

THE report of Monsieur Kelsch, of the permanent Vaccine Commission of the French Academy of Medicine, is very flattering to the Lister Institute at Chelsea. He says he considers it the best installation of all that he visited during his comprehensive tour of inspection, and in fact he believes that it is perfect.

THE outbreak of typhoid in the Rhondda Valley is of a serious nature. The number on November 14th was 150. The epidemic is traced to the water supply. Strict measures are being taken to prevent any further contagion from this source. The Isolation hospital has not sufficient accommodation for the demands made upon it, and many of the patients are being treated in their own homes.

MR. R. STEWART JOHNSON has been appointed secretary at the Hospital for Sick Children, Great Ormond Street. Mr. Stewart Johnson is a barrister and a graduate of Cambridge. For the last two years he has been engaged in an honorary capacity in the Secretarial Department of the East London Hospital for Children, where he has performed all the duties connected with it under the able and experienced guidance of Mr. Thomas Hayes. We understand that he will enter upon his new duties immediately. Mr. Stewart Johnson has those social qualities which should especially fit him for the position he is about to occupy.

